# A Dual-Signal Ratiometric Optical Sensor Based on Natural Pine Wood and Platinum(II) Octaethylporphyrin with High Performance for Oxygen Detection

**DOI:** 10.3390/s25133967

**Published:** 2025-06-26

**Authors:** Zhongxing Zhang, Yujie Niu, Hongbo Mu, Jingkui Li, Jinxin Wang, Ting Liu

**Affiliations:** Department of Physics, Northeast Forestry University, Harbin 150040, China; cqxnh@nefu.edu.cn (Z.Z.); niuyujie@nefu.edu.cn (Y.N.); mhb506@nefu.edu.cn (H.M.); li_jing_kui@nefu.edu.cn (J.L.); wangjinxin@nefu.edu.cn (J.W.)

**Keywords:** pine wood, PtOEP, oxygen-sensing, PDMS, optical parameter

## Abstract

Optical oxygen sensors have attracted considerable attention owing to their high sensitivity, rapid response, and broad applicability. However, their test results may be affected by fluctuations in the pump light source and instability of the detection equipment. In this study, the intrinsic luminescence of pine wood was utilized as the reference signal, and the luminescence of platinum(II) octaethylporphyrin (PtOEP) was employed as the oxygen indication signal, to fabricate a dual-signal ratiometric oxygen sensor PtOEP/PDMS@Pine. The ratio of the luminescence of pine wood to that of PtOEP was defined as the optical parameter (*OP*). *OP* increased linearly with oxygen concentration ([*O*_2_]) in the range of 10–100 kPa, and a calibration curve was obtained. The sensor exhibits excellent anti-interference capabilities, effectively resisting fluctuations from laser sources and detection equipment. It also displays stable hydrophobicity with a contact angle of 118.3° and maintains excellent photostability under continuous illumination. The sensor exhibited long-term stability within 90 days and robust recovery performance during cyclic tests, wherein the response time and recovery time were determined to be 1.4 s and 1.7 s, respectively. Finally, the effects of temperature fluctuations and photobleaching on the sensor’s performance have been effectively corrected, enabling accurate oxygen concentration measurements in complex environments.

## 1. Introduction

Oxygen is a critical component that sustains life on Earth and plays an essential role in numerous natural and industrial processes. Accurate monitoring of oxygen levels is an indispensable and critical technique in modern society, directly impacting life safety, industrial production efficiency, and the maintenance of ecological balance [1,2,3,4,5]. The optical oxygen detection method based on the luminescence quenching principle has garnered significant attention due to its advantages, including a rapid response, no consumables, and high sensitivity [6,7,8,9,10]. However, the detection results are frequently influenced by fluctuations in the pump light source and equipment stability, and its relatively high cost further restricts the large-scale application of this method [11,12,13]. Consequently, developing an optical oxygen detection method with enhanced anti-interference capability and reduced cost is crucial for its wide-ranging application.

The dual-signal ratiometric method can effectively address the limitations of traditional optical methods [14,15,16,17]. By employing an oxygen-insensitive signal as the reference and an oxygen-sensitive signal as the indication, and correlating the ratio of these two signals with oxygen concentration, this can effectively mitigate the impact of pump light source fluctuations and detection equipment instability during testing. Typically, oxygen-insensitive signals are generated by molecules whose luminescence is unaffected by oxygen. By incorporating these molecules, along with oxygen-sensitive indicators, into the supporting matrix, optical oxygen sensors are developed [18,19,20,21]. The supporting matrix is constructed using oxygen-selective membranes [22,23,24,25,26]. However, the reference signal molecules and indicator signal molecules demonstrate a degree of selectivity when binding to the supporting matrix; the molecules cannot randomly incorporate into any matrix material. This limitation, in turn, constrains the diversity of sensor types. Additionally, due to the relatively complex preparation process, the fabricated sensors typically fail to exhibit fully consistent performance.

Wood is a naturally renewable organic material and serves as an important green resource, exhibiting stable chemical properties and excellent mechanical performance [27,28]. Notably, wood emits strong luminescence signals under visible light excitation, and the luminescence is unaffected by environmental oxygen [29]. Not only that, its porous structure enables the rapid diffusion of oxygen molecules. Additionally, the porous structure facilitates the attachment of a wide variety of molecules [30], imposing no restrictions on their selective binding. The combination of any molecule with wood can be realized via a physical impregnation process, thereby simplifying the preparation procedure. Based on these characteristics, wood can serve simultaneously as both the reference signal source and the supporting matrix for oxygen sensors. However, its potential applications in oxygen sensors remain unexplored. Among various types of wood, pine wood is widely distributed, low-cost, and easy to process, making it a suitable candidate for use as a component in oxygen sensors [31,32,33]. By integrating it with oxygen-sensitive molecules, a highly adaptable, cost-effective, and robustly anti-interference optical oxygen sensor can be developed through a simplified preparation procedure. However, pine wood has a natural tendency to absorb moisture, leading to mold growth and deformation [34,35]. This can substantially compromise the testing performance of the sensor and diminish its long-term storage stability. Therefore, before utilizing pine wood as a component of the oxygen sensor, its hydrophobic properties must be enhanced.

Polydimethylsiloxane (PDMS) exhibits hydrophobicity and oxygen permeability, is easy to process, low-cost, environmentally friendly, and chemically stable. It is commonly used as the supporting matrix for optical oxygen sensors [36,37,38]. PDMS exhibits no luminescence under visible light excitation [39]. Based on these properties, PDMS is appropriate to be used to enhance the hydrophobicity of pine wood when it serves as a component in optical sensing systems without interfering with extra optical signals.

Platinum and palladium porphyrins are frequently utilized as oxygen-sensitive molecules in optical oxygen sensors owing to their high sensitivity to oxygen and superior stability [40,41]. In this study, pine wood was selected as the reference signal source and supporting matrix, while platinum(II) octaethylporphyrin (PtOEP) served as the oxygen indicator molecule. PDMS was incorporated to enhance the hydrophobic properties of pine wood, and finally, a low-cost, highly interference-resistant optical oxygen sensor PtOEP/PDMS@Pine was fabricated via a physical impregnation process. The morphological features of the sensor, the distribution of PtOEP, and the infiltration depth of PDMS within the sensor matrix were systematically studied. The optimal concentration of PtOEP within the sensor was systematically investigated, and the impact of different preparation methods on sensor performance was comprehensively analyzed to achieve performance optimization. Based on the sensor, a quantitative relationship between the optical parameter (*OP*) and oxygen concentration ([*O*_2_]) was established, and a calibration curve was obtained. The ability of the sensor to withstand the fluctuations in pump light source and detection equipment was accessed. Additionally, its hydrophobic characteristics, response and recovery performance, and long-term stability were comprehensively evaluated. The impact of humidity on sensor performance was systematically assessed. Moreover, the effects of photobleaching and temperature fluctuations on the sensor performance were systematically studied, and the drift in the calibration curve induced by these factors was effectively compensated for. The PtOEP/PDMS@Pine sensor designed in this study is expected to demonstrate extensive application potential for oxygen measurement.

## 2. Materials and Methods

### 2.1. Materials

Platinum(II) octaethylporphyrin (PtOEP) was obtained from Shanghai Macklin Bio-chemical Technology Co., Ltd. (Shanghai, China). Toluene was obtained from Xilong Reagent Co., Ltd. (Guangzhou, China). Polydimethylsiloxane (PDMS) was purchased from DOW Chemicals Co., Ltd. (Guangzhou, China). Pine wood originated from Harbin Haicheng Wood Processing Plant (Harbin, China).

### 2.2. Preparation of the PtOEP/PDMS@Pine Sensor

The PtOEP/PDMS@Pine sensor in this study was fabricated through a straightforward process involving physical impregnation. Specifically, PtOEP was dissolved in toluene to yield a PtOEP solution with a concentration of 1 mM. Flaky-like pine wood with a dimension of 10 × 10 × 1 mm^3^ (length × width × thickness) and a density of 0.50 g/cm^3^ was selected as the wood matrix. The wood matrix was immersed in the PtOEP/toluene solution (10 wt%) for 10 min and subsequently dried for 1 h in room temperature. The prepared wood is encapsulated with the PDMS/toluene solution via the physical impregnation process, followed by drying for 48 h at room temperature to finalize the preparation. All procedures were conducted in a darkroom environment.

### 2.3. Instruments and Characterization

The morphological characteristics of untreated pine wood and the PtOEP/PDMS@Pine sensor were investigated using an ultra-high resolution field emission scanning electron microscope (SEM, Apreo 2c, Thermo Fisher, Waltham, MA, USA), equipped with an energy dispersive spectrometer (EDS) for elemental mapping of the sensor’s cross-section. The hydrophobicity of the sensor was characterized using a contact angle measuring instrument (SDC–350, SINDIN, Dongguan, China). The contact angle of the sensor was monitored for 60 s to investigate the stabilization of its hydrophobicity. Additionally, the contact angle of pine wood and PtOEP-treated pine wood were monitored for comparison.

In this study, the photoluminescence spectra of PtOEP, pine wood, PDMS, and the PtOEP/PDMS@Pine sensor were measured to investigate the fundamental optical characteristics of the sensor. A 405 nm diode laser (Shanghai Oxlasers Electronics Co., Ltd., Shanghai, China) served as the light source with a power density of 1.5 mW/cm^2^. The luminescence signal was collected by a fiber optic spectrometer (USB2000, Ocean Optics, Orlando, FL, USA). The photoluminescence spectra of different pine wood samples and those measured by various sensors were analyzed to investigate the differences in luminescence characteristics among the sensors across different samples.

To investigate the relationship between *OP* and oxygen concentration, the photoluminescence spectra of the sensor under various oxygen concentrations were measured. Pure oxygen and pure nitrogen were mixed via mass flow controllers (Sevenstar D07-19B, BeiJing Sevenstar Flow Co., Ltd., Beijing, China) to generate mixed gases with precise oxygen concentrations. These gases were then introduced into a sealed gas chamber equipped with an optical detection window and a non-return valve to ensure stable testing conditions. The sample placed at the optical window was irradiated using a 405 nm laser, and its luminescence signal was collected by an optical fiber spectrometer (USB2000). The calibration curve was obtained by performing linear fitting analysis on the *OP* values measured at various oxygen concentrations. Pine wood samples were impregnated in PtOEP/toluene solutions at varying concentrations to fabricate sensors with differing PtOEP contents. The optimal concentration of the PtOEP/toluene solution for sensor preparation was determined by analyzing the calibration curves of the resulting sensors; the impact of preparation methods on sensor performance was further investigated. Two distinct preparation approaches were compared in the experiment: (i) PtOEP and PDMS were homogeneously mixed in toluene, followed by immersing the pine wood in the mixture for 10 min, then drying it for 48 h; and (ii) the pine wood was first immersed in a PtOEP/toluene solution for 10 min and dried for 1 h, followed by hydrophobic treatment with PDMS, and finally dried for 48 h to obtain the final sensor. The performance of the pine wood sensor, which was impregnated solely in the PtOEP/toluene solution without undergoing hydrophobic treatment with PDMS, was also compared and analyzed.

To evaluate the anti-interference capability of the sensor under fluctuations in detection equipment and laser power density, *OP* values were measured 30 times at laser power densities of 0.5 mW/cm^2^, 1 mW/cm^2^, and 1.5 mW/cm^2^, respectively, when the oxygen concentration environment was fixed at 10 kPa. The reference signal (*A*_1_) and indication signal (*A*_2_) were also measured and analyzed for comparison. The optical stability of the sensor was assessed by quantifying the integrated intensity of its photoluminescence spectra in the wavelength ranges of 477–523 nm (denoted as *A*_1_) and 650–745 nm (denoted as *A*_2_) during a 1 h continuous exposure to a 405 nm light source (with a laser power density of 1.5 mW/cm^2^) under oxygen-free conditions. The response time of the sensor was assessed by monitoring the change in luminescence intensity at a 650 nm wavelength during the transition from a pure nitrogen atmosphere to a pure oxygen atmosphere under 405 nm laser irradiation. The response time was defined as the time required for the luminescence intensity to decrease to 95% of its initial value upon switching from a pure nitrogen atmosphere to a pure oxygen atmosphere; the recovery time was defined as the time needed for the luminescence intensity to return to 95% of its initial value when switching back from pure oxygen to pure nitrogen. This process was repeated three times to evaluate the recovery performance of the sensor. The gas chamber used for measuring the response time had a volume of 0.036 L, with a gas flow rate of 0.3 L/s. Consequently, it took 0.12 s for the gas to be fully replaced. A thermal stage capable of simultaneous temperature control and ventilation was employed to investigate the influence of temperature on sensor performance (LTS120, Linkam Scientific Instruments Ltd., Lincoln, UK). To investigate the influence of humidity on the sensor, its *OP* values were measured in gas environments with humidity levels of 50%, 70%, and 90%, under oxygen partial pressures of 10 kPa, 30 kPa, and 50 kPa. The long-term stability of the sensor was assessed by measuring its *OP* values in environments with oxygen concentrations of 10 kPa, 50 kPa, and 90 kPa over a 90-day placement period.

## 3. Results and Discussion

### 3.1. The Morphology Characteristics of the PtOEP/PDMS@Pine Sensor

**Figure 1:**
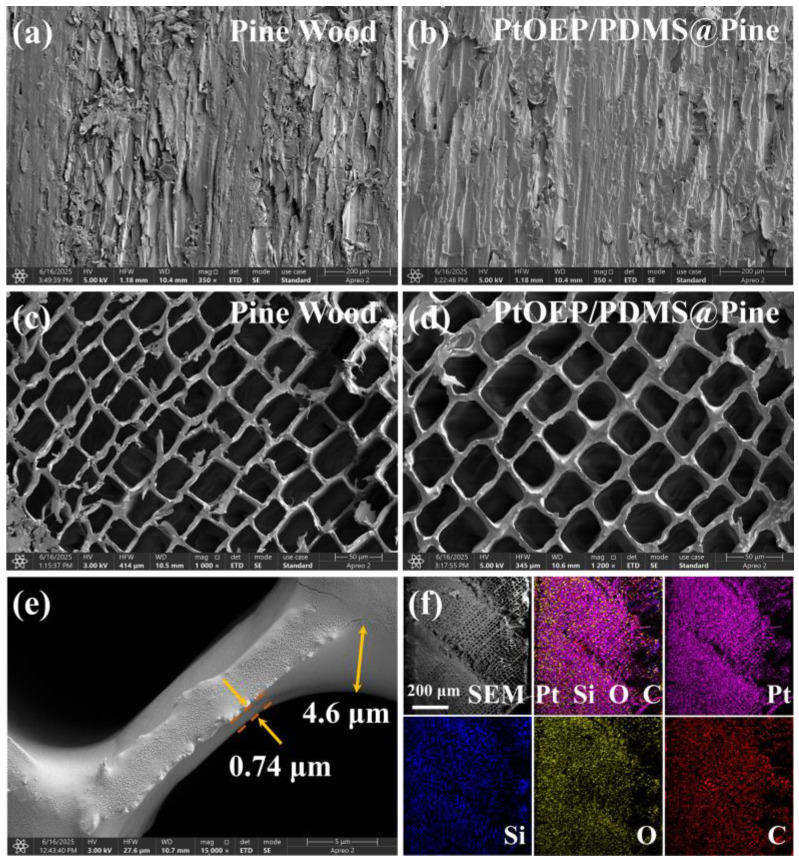
SEM of pine wood and PtOEP/PDMS@Pine. (**a**,**b**) Tangential sectional morphology; (**c**,**d**) cross-sectional morphology; (**e**) thickness of PDMS on pine wood; (**f**) element mapping of Pt, Si, O, and C in a cross-section of PtOEP/PDMS@Pine.

The microscopic morphological characteristics of pine wood and the PtOEP/PDMS@Pine sensor were investigated, as shown in Figure 1. The tangential section of untreated pine wood shown in Figure 1a exhibited relatively high surface roughness. In contrast, the PtOEP/PDMS@Pine sensor (Figure 1b) showed a significantly smoother and more uniform surface, indicating that the coating method employed effectively enhanced the surface quality of the wood substrate. Moreover, cross-sectional morphologies of the pine wood and treated sample were comparatively assessed (Figure 1c,d). The pine wood exhibited a typical regular polygonal porous structure, characterized by interconnected pores with uniform distribution and an overall calculated porosity of approximately 63.2%. After impregnation treatment with PtOEP and PDMS, the pores remained open and unobstructed, and their internal pores became noticeably smoother and more rounded, demonstrating that the coating did not adversely affect the inherent porous structure, thus ensuring effective diffusion pathways for oxygen molecules. This is a critical factor for oxygen-sensing applications. Furthermore, the thickness of the PDMS coating layer was precisely measured, as shown in Figure 1e. The PDMS layer exhibited excellent conformity with the wood surface, with a coating thickness ranging from 0.74 μm to 4.6 μm and an average thickness of approximately 1.9 μm. This result confirms the uniformity and controllability of the developed coating process. To determine the infiltration depths of PtOEP and PDMS in the pine wood, the sensor was sectioned at its midpoint, and elemental mapping of the cross-section was analyzed, including C, O, Si, and Pt. Notably, Si originated exclusively from PDMS, while Pt stemmed from PtOEP. The results are presented in Figure 1f. They indicate that Pt and Si are homogeneously distributed across the entire cross-section of the pine wood matrix, thereby confirming that PtOEP and PDMS are uniformly dispersed within the whole microstructure of the pine wood. Consequently, the effective functional layer thickness in the sensor corresponds to the thickness of the pine wood matrix (1 mm). Such homogeneous elemental distribution is anticipated to significantly enhance both the reliability and stability of the material in practical oxygen-sensing applications.

### 3.2. The Hydrophobicity of the PtOEP/PDMS@Pine Sensor

**Figure 2:**
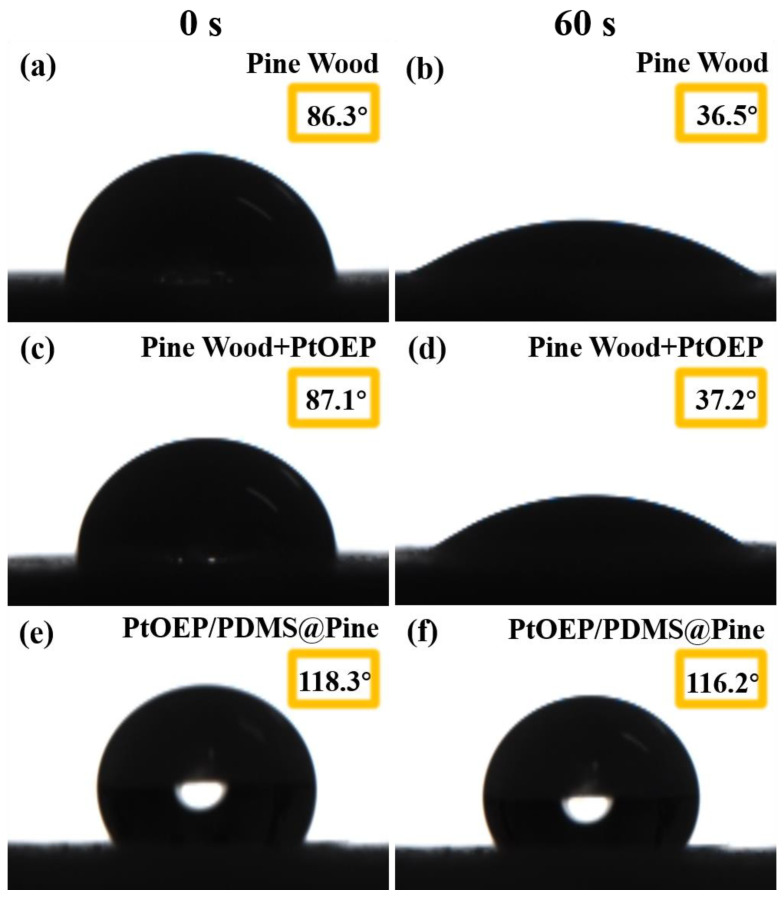
The water contact angle of pine wood (**a**,**b**); Pine wood combined with PtOEP (**c**,**d**); PtOEP/PDMS@Pine sensor (**e**,**f**) monitored at 0 s and 60 s.

In this work, the hydrophobic properties of the sensor are enhanced to mitigate the adverse effects of pine wood’s hygroscopic nature on sensor performance. The hydrophobic property of the sensor was characterized, and the untreated pine wood and pine wood only treated with PtOEP were also measured for comparison. The water contact angle of all samples was observed over a 60 s period, and the results are shown in Figure 2. It can be observed that the water contact angle of the untreated pine wood decreased rapidly from an initial value of 86.3° to 36.5° within 60 s. During the experiment, it was clearly observed that water droplets quickly penetrated into the wood upon contact with its surface. Similarly, pine wood treated only with PtOEP exhibited the same trend, with its contact angle decreasing from 87.1° to 37.2° within 60 s. In contrast, the PtOEP/PDMS@Pine sensor displayed significantly different characteristics. Its water contact angle started at 118.3° at 0 s and only slightly decreased to 116.2° after 60 s, remaining stable during the observation. These findings indicate that PDMS successfully improved the hydrophobic properties of the pine wood, thereby enhancing its moisture resistance and effectively mitigating the adverse effects of pine wood’s hygroscopic nature on the sensor.

### 3.3. The Optical Properties of the PtOEP/PDMS@Pine Sensor

**Figure 3:**
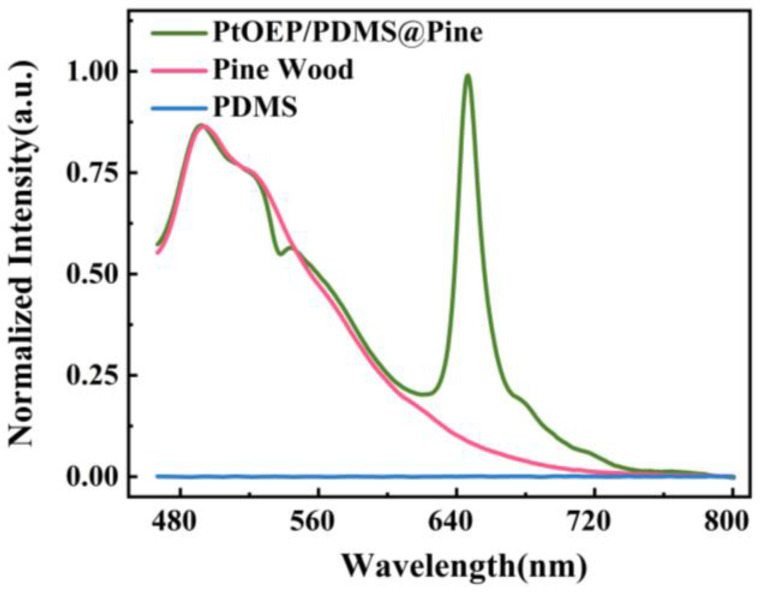
The photoluminescence of PDMS, pine wood, and the PtOEP/PDMS@Pine sensor.

In this study, pine wood was selected as both the supporting matrix and the reference signal source simultaneously. It was combined with the oxygen-sensitive indicator PtOEP through a physical impregnation process, followed by hydrophobic modification using PDMS. Consequently, the PtOEP/PDMS@Pine sensor was successfully fabricated. To explore the optical properties of the sensor, the photoluminescence spectrum was measured. Additionally, the photoluminescence spectra of pine wood and PDMS were also measured for comparison, as illustrated in Figure 3. From the figure, it is evident that PDMS exhibits no luminescence within the measured wavelength range, whereas pine wood displays a broad luminescence spanning 466 nm to 720 nm. Additionally, the luminescence range of the PtOEP/PDMS@Pine sensor spans 466 nm to 745 nm, with its spectral characteristics comprising both the intrinsic luminescence of pine wood and PtOEP. Specifically, in the 600 nm to 750 nm range, the luminescence of the sensor aligns with that of PtOEP, while in the 466 nm to 600 nm range, the luminescence largely overlaps with that of pine wood. Notably, compared with pine wood, there is a distinct “depression” in the luminescence of the sensor centered at 540 nm. Comparing with the absorption spectrum of PtOEP [42], it is confirmed that the depressed peak in the photoluminescence spectrum of the sensor corresponds to the Q band absorption peak of PtOEP centered at 540 nm. The above results demonstrate that the physical impregnation preparation process effectively preserves the intrinsic optical properties of both the pine wood and PtOEP.

**Figure 4:**
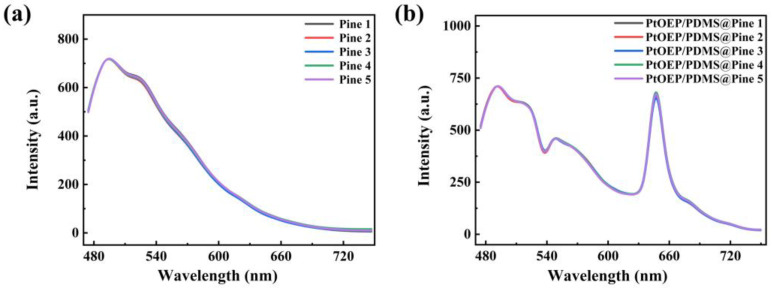
(**a**) The photoluminescence spectra of different pine wood samples; (**b**) various sensors.

Due to variations in the growth environment, tree age, wood type (e.g., sapwood vs. heartwood), and physiological state, the luminescence characteristics of pine wood can differ significantly, this may lead to considerable variations among the fabricated sensors. Therefore, the photoluminescence spectra of different pine wood samples and various sensors were investigated to thoroughly examine the optical properties of the sensors, as shown in Figure 4. Figure 4a presents the photoluminescence spectra of various pine wood samples. It is evident that the profile and intensity of the photoluminescence spectra of untreated pine wood almost remain unchanged. Figure 4b illustrates the photoluminescence spectra of different sensors. As shown in the figure, the spectral profile and intensity are consistent across the sensors, indicating that the luminescence characteristics of pine wood remain stable in different sensors. This consistency arises because all the wood matrix materials used in the experiment were sourced from the same piece of wood, ensuring uniformity in their primary physical properties and chemical composition. To ensure the consistency of the optical properties of the fabricated sensors, pine wood with uniform physical characteristics, such as density, porosity, and anatomical location, should be selected as the matrix material.

### 3.4. The Establishment of the Calibration Curve Based on the PtOEP/PDMS@Pine Sensor

**Figure 5:**
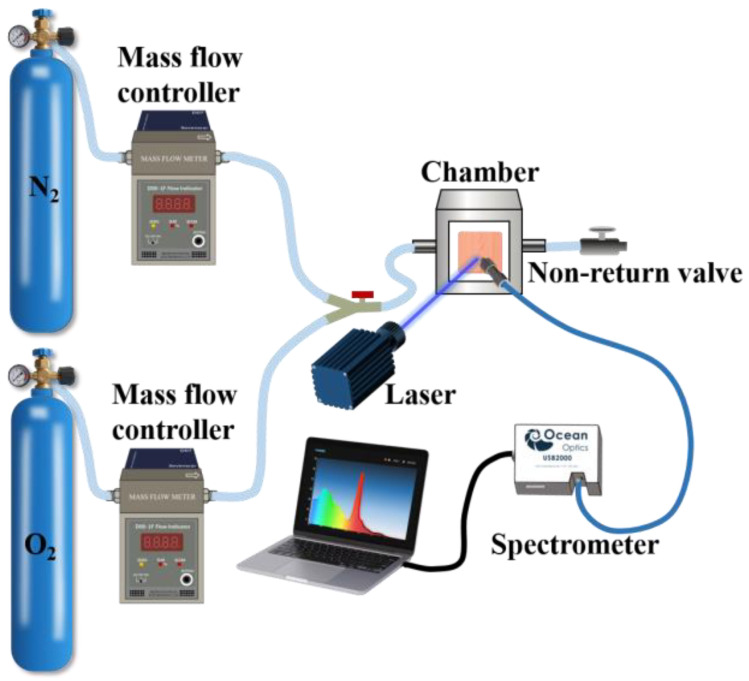
The setup for photoluminescence measurement in various oxygen concentrations.

To investigate the characteristics of the sensor to oxygen, the photoluminescence spectra of the sensor under different oxygen environments were measured using the setup presents in Figure 5.

**Figure 6:**
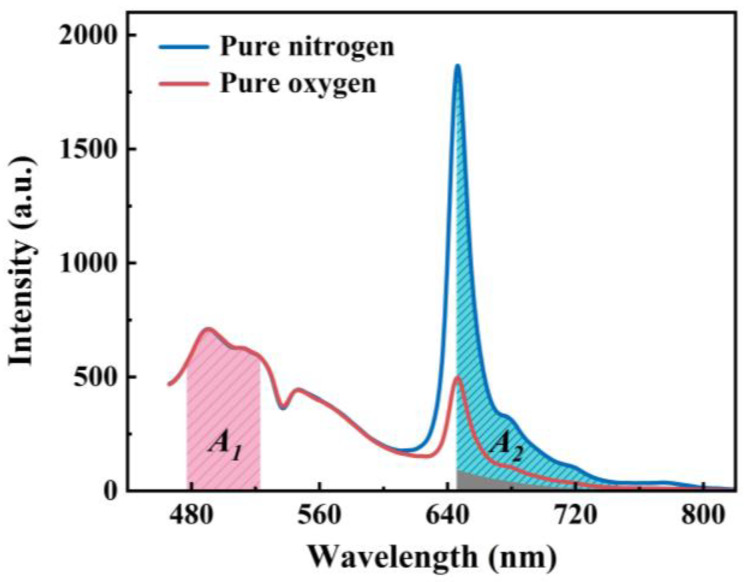
Photoluminescence spectra of the PtOEP/PDMS@Pine sensor in pure nitrogen and pure oxygen environments; *A*_1_, *A*_2_ and the gray area represent the integration intervals of the reference signal, the oxygen indication signal, and the overlapping spectral region between pine wood and PtOEP, respectively.

The photoluminescence spectra of the sensor in pure nitrogen and pure oxygen atmospheres are exhibited in Figure 6. It was observed that the profile of the photoluminescence spectra remained consistent in both pure nitrogen and pure oxygen atmospheres. Within the wavelength range of 475–600 nm, the luminescence intensity did not vary with changes in oxygen concentration. This is attributed to the intrinsic luminescence of the pine wood, whose luminescence intensity only depends on the excitation light power density. In contrast, within the wavelength range of 600–745 nm, the luminescence intensity decreased significantly with increasing oxygen content. This emission originates from the inherent luminescence of PtOEP molecules, whose intensity is influenced by both the excitation light power density and oxygen. Based on the aforementioned characteristics, the luminescence of pine wood can serve as the reference signal, while the luminescence of PtOEP can function as the indicator signal, enabling the construction of a ratiometric oxygen sensor. As illustrated in Figure 3, analysis of the spectral properties of the sensor components indicates that pine wood displays a broad luminescence band, which partially overlaps with the entire emission peak of PtOEP. Furthermore, this overlapping region diminishes progressively with increasing wavelengths. To minimize the impact of the overlapping signals on the accuracy of sensor measurements, the spectral integral intensity was adopted as the signal. This approach effectively reduces the uneven variations in the two signals caused by environmental factors, thereby enhancing the anti-interference capability and stability of the sensor. Furthermore, the integral intensity of the right half peak of PtOEP (650–745 nm) was selected to be the indication signal to avoid overlap with the reference signal as much as possible. However, within this luminescence wavelength range of PtOEP, there is still an overlap with the luminescence signal of the wood. Given that the luminescence of pine wood in the sensor is independent of the oxygen concentration, it follows that the integral intensity of the overlapping region between pine wood and PtOEP remains unchanged. Based on these observations and analyses, *A*_1_ is defined as the integral area of the photoluminescence spectrum of the PtOEP/PDMS@Pine sensor within the wavelength range of 477–523 nm, which can serve as the reference signal, while *A*_2_ is defined as the difference between the integral intensity of the right half-peak of PtOEP and the integral intensity of the overlapping region between PtOEP and the luminescence of pine wood within the wavelength range of 650–745 nm, and can function as the indication signal. The ratio of *A*_1_ to *A*_2_ is designated as the optical parameter (*OP*), which correlates exclusively with the oxygen concentration ([*O*_2_]).

(1) $$OP=\frac{A_{1}}{A_{2}}=f^{-1}([O_{2}])$$

From the above relationship, it can be seen that an increase in the selection range of *A*_1_ will lead to enhanced sensitivity to oxygen. Consequently, this explains why the maximum spectral range integral of pine wood, which is unaffected by the oxygen-sensitive signal, is selected as the reference signal.

**Figure 7:**
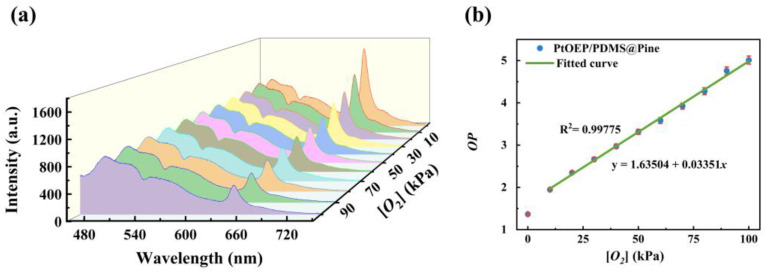
(**a**) Photoluminescence spectra of the PtOEP/PDMS@Pine sensor in various oxygen concentrations; (**b**) *OP* values versus oxygen concentrations.

To further investigate the relationship between *OP* and oxygen concentration, the photoluminescence spectra of the sensor were measured under varying oxygen concentrations (as shown in Figure 7a). Through calculation, the *OP* values corresponding to different oxygen concentrations were determined (as shown in Figure 7b). It is evident that as the oxygen concentration increases, the *OP* value gradually rises. Within the oxygen concentration range of 10–100 kPa, the *OP* value exhibits a linear growth trend, which can be described by the Stern–Volmer equation [43].

(2) $$\frac{I_{P0}}{I_{P}}=1+K_{\mathrm{SV}}[O_{2}]$$

where *I*_P0_ is the phosphorescence intensity in the absence of oxygen (equivalent to *A*_1_), *I*_P_ is the phosphorescence intensity in the presence of oxygen (equivalent to *A*_2_), and [*O*_2_] is the oxygen concentration of the substance to be measured. *K*_SV_ is the constant for the phosphorescence to be quenched by oxygen, which represents the sensitivity of the oxygen sensor. Subsequently, by performing linear fitting on the data, a calibration curve based on this sensor was successfully established, as follows.

(3) $$OP=1.64+0.03[O_{2}]$$

Based on this relationship, the oxygen concentration can be precisely determined through the measurement of the optical signal of the sensor.

### 3.5. The Impact of PtOEP Concentration and Preparation Methodology on the Performance of the PtOEP/PDMS@Pine Sensor

**Figure 8:**
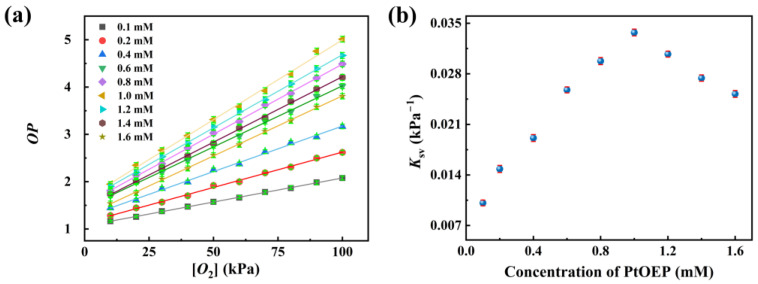
(**a**) The calibration curves of the sensors containing various PtOEP concentrations; (**b**) *K*sv values for the sensors of different PtOEP concentrations.

To optimize the performance of the sensor, the effects of the PtOEP concentration and the sensor preparation approach on its performance were studied. Pine wood was immersed in PtOEP/toluene solutions with varying concentrations to fabricate sensors containing different PtOEP contents. The calibration curves of the sensors containing various PtOEP concentrations were obtained, as presented in Figure 8a. It shows that *OP* values of the sensors containing variable PtOEP concentrations linearly increased with increasing oxygen concentration within the oxygen partial pressure range of 10 kPa to 100 kPa. By performing linear fitting on the experimental data, the *K*sv values for the sensors at different PtOEP concentrations were obtained, as shown in Figure 8b. It can be observed that as the PtOEP concentration increases, the *K*sv value initially rises and then decreases. The maximum *K*sv of 0.36 was achieved at a PtOEP concentration of 1 mM. Therefore, a 1 mM PtOEP/toluene solution is determined to be the optimal concentration for sensor preparation. The pine wood exhibits a porous structure with numerous large pores, providing ample attachment sites for PtOEP both on its surface and within its interior. This facilitates the uniform distribution of PtOEP with a high concentration. In the subsequent experiments, all sensors were fabricated using this PtOEP solution concentration.

**Figure 9:**
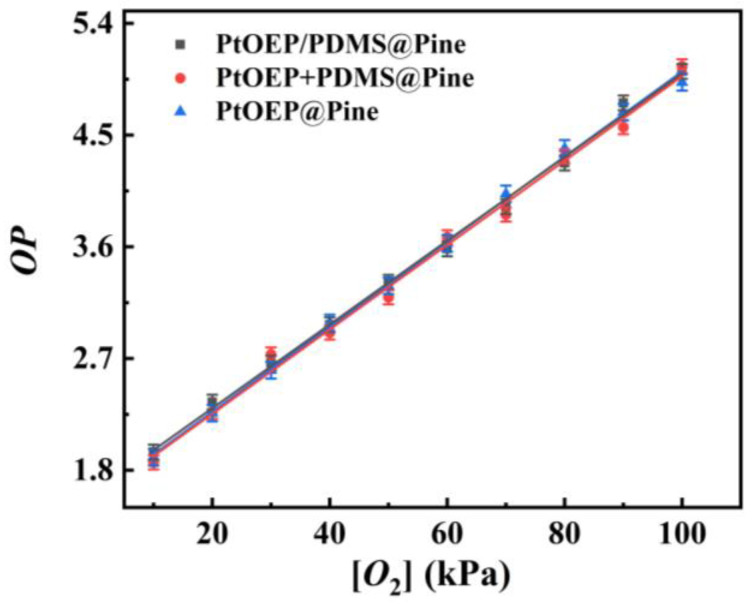
The calibration curves of the sensors by different approaches.

In addition, the impact of preparation methods on sensor performance was further investigated. Two distinct preparation approaches were compared in the experiment: (i) PtOEP and PDMS were homogeneously mixed in toluene, followed by immersing the pine wood in the mixture for 10 min, then drying it for 48 h; and (ii) the pine wood was first immersed in a PtOEP/toluene solution for 10 min and dried for 1 h, followed by hydrophobic treatment with PDMS, and finally dried for 48 h to obtain the final sensor. By measuring the *OP* values of the three sensors under varying oxygen concentrations, calibration curves were generated (as shown in Figure 9). The results demonstrated that the calibration curves obtained from the two preparation methods were nearly identical. Based on the observations and analysis of the experimental process, it can be concluded that the consistency in sensor performance between the two preparation methods can be attributed to the relatively low concentration of the PDMS solution (which is dilute), combined with the uniform distribution of PtOEP on both the surface and interior of the wood. The extended drying time during the hydrophobic treatment enables PtOEP molecules within the wood to fully dissolve into the PDMS solution. Consequently, the performance of the sensor prepared layer by layer is consistent with that of the sensor prepared by immersing the wood in the PtOEP/PDMS mixed solution.

Meanwhile, the performance of the pine wood sensor, which was impregnated solely in the PtOEP/toluene solution without undergoing hydrophobic treatment with PDMS, was also compared and analyzed. By comparing its calibration curve with those obtained using methods (i) and (ii), it is evident that the calibration curve of the pine wood sensor treated solely with PtOEP is consistent with that of the PDMS-coated sensor. This consistency can be attributed to the porous structure of the wood, which promotes rapid oxygen diffusion within the material and ensures that the oxygen-sensitive molecules uniformly distributed throughout the pine wood can promptly interact with oxygen molecules. Furthermore, PDMS, an exceptional oxygen-selective material, forms a thin coating on the wood surface with a thickness of approximately 1 μm. This coating neither impedes oxygen diffusion nor compromises the interaction between oxygen-sensitive molecules and oxygen molecules, thereby ensuring their sufficient interaction.

### 3.6. The Anti-Interference Capability of the PtOEP/PDMS@Pine Sensor

**Figure 10:**
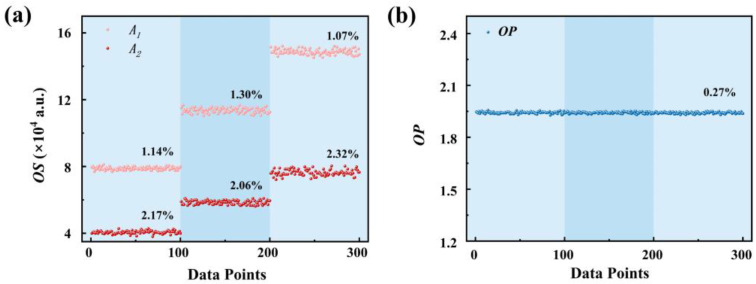
The values of (**a**) *A*_1_, *A*_2_; and (**b**) *OP* of the PtOEP/PDMS@Pine sensor at excitation light power densities of 0.5 mW/cm^2^, 1.0 mW/cm^2^, and 1.5 mW/cm^2^.

Theoretically, the dual-signal ratiometric method can effectively eliminate the influence of pump light source fluctuations and detection equipment instability on the measurement results compared with a single optical signal. In this study, the anti-interference capability of the dual-signal PtOEP/PDMS@Pine sensor was systematically evaluated. The sensor was exposed to a 405 nm laser in a 10 kPa oxygen atmosphere, and the OP values were continuously measured 30 times under excitation light power densities of 0.5 mW/cm^2^, 1.0 mW/cm^2^, and 1.5 mW/cm^2^, respectively. The direct optical signals (*OSs*) *A*_1_ and *A*_2_ were also measured for comparison. Here, the fluctuation was quantitatively assessed using the ratio of the standard deviation to the mean value of the data, and the results are shown in Figure 10. It is evident that the fluctuations of *A*_1_ (~1%) and *A*_2_ (~2%) are significantly larger than those of the *OP* values (~0.3%) under each laser power density condition. Additionally, as the excitation light power density increases, the values of *A*_1_ and *A*_2_ increase correspondingly, whereas the *OP* value remains nearly constant. These results demonstrate that the dual-signal ratio method exhibits superior stability compared to a single optical signal and can effectively mitigate the effects of excitation light fluctuations and detection equipment instability on the measurement results. The dual-signal PtOEP/PDMS@Pine sensor developed in this work demonstrates superior anti-interference capability during the measurement process, ensuring precise determination of the oxygen concentration in complex and fluctuating conditions.

### 3.7. The Response Time and Recovery Performance of the PtOEP/PDMS@Pine Sensor

**Figure 11:**
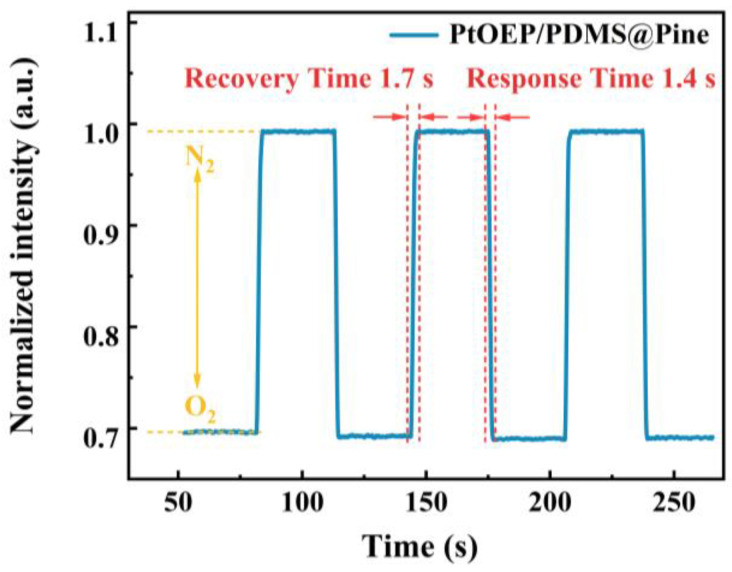
The luminescence intensity of the PtOEP/PDMS@Pine sensor monitored at 650 nm in atmospheres switching between aerobic and anaerobic.

Response time and recovery performance are critical factors that significantly influence the applicable and reliability of sensors, serving as two core indicators for evaluating sensor performance [44]. A fast response time ensures timely acquisition of results during the testing process, thereby minimizing errors caused by delays. Good recovery performance enables the sensor to provide stable and reliable results during continuous monitoring. In this study, the response time and recovery performance of the sensor were assessed by monitoring the changes in luminescent intensity during the switching process between pure oxygen and anaerobic atmospheres. As illustrated in Figure 11, upon switching the atmosphere from pure oxygen to pure nitrogen, the luminescence intensity of the sensor increased rapidly. Conversely, when the atmosphere was switched back from pure nitrogen to pure oxygen, the light intensity promptly decreased to its initial level. The response time is defined as the time required for the luminescent intensity of the sensor to reach 95% of its initial value when transitioning from an anaerobic to an aerobic environment. The recovery time is defined as the time required for the luminescent intensity to reach 95% of its initial value when transitioning from an aerobic to an anaerobic environment. The fast response time and recovery time were determined to be 1.4 s and 1.7 s, respectively. During the three-cycle tests, the sensor exhibited remarkable recoverability. The results demonstrate that the sensor is capable of accurately measuring oxygen concentration in a dynamically fluctuating environment with high efficiency.

### 3.8. The Optical Stability of the PtOEP/PDMS@Pine Sensor

**Figure 12:**
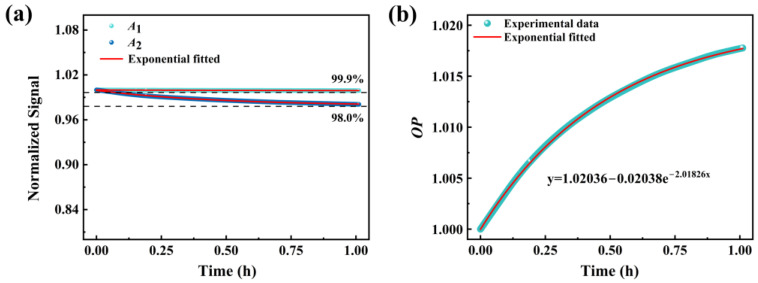
(**a**) *A*_1_ and *A*_2_ signals under irradiation of a 405 nm laser monitored for 1 h; (**b**) *OP* values at various light exposure times.

For optical oxygen sensors, the oxygen indicator molecules always undergo irreversible photochemical reactions under continuous illumination, leading to the destruction of their chemical structure [45]. This phenomenon results in a gradual weakening of the sensor’s signal strength, which in turn reduces the sensitivity and signal-to-noise ratio of the sensor. In this study, the optical stability of the PtOEP/PDMS@Pine sensor was evaluated. The application signals of the sensor consist of two components: the reference signal *A*_1_, which originates from the intrinsic luminescence of the wood, and the oxygen-sensitive signal *A*_2_, which arises from the luminescence of PtOEP. Variations in the luminescence intensity of both signals can induce drift in the sensor’s calibration curve. To address this issue, the intensity of both signals under continuous illumination were measured in a pure nitrogen environment, as depicted in Figure 12a. From the figure, it is evident that both *A*_1_ and *A*_2_ exhibit decreases under continuous illumination. The photobleaching rates are determined to be 0.01%/h (*A*_1_) and 0.20%/h (*A*_2_), respectively. This result demonstrates that both the reference and oxygen-sensitive signals are subject to photobleaching effects. The asynchronous decay rates of *A*_1_ and *A*_2_ can lead to calibration curve drift, thereby compromising measurement accuracy. Consequently, the original calibration curve must be corrected to ensure accurate measurements. The *OP* values at various light exposure times were calculated based on the *A*_1_ and *A*_2_ values presented in Figure 12a. Subsequently, an exponential fitting method was applied to derive the growth curve of *OP* as a function of light exposure time (Figure 12b), which is illustrated below.

(4) $$y=1.02-0.02e^{-2t}$$

Based on this equation, the original calibration curve was corrected, yielding a new curve that incorporates the illumination time factor. By measuring the specific value of *OP*(*t*) at time *t*, the precise oxygen concentration can be accurately determined, as shown in the following formula.

(5) $$\frac{OP(t)}{1.02-0.02e^{-2t}}=1.64+0.03[O_{2}]$$

### 3.9. The Long-Term Stability of the PtOEP/PDMS@Pine Sensor

**Figure 13:**
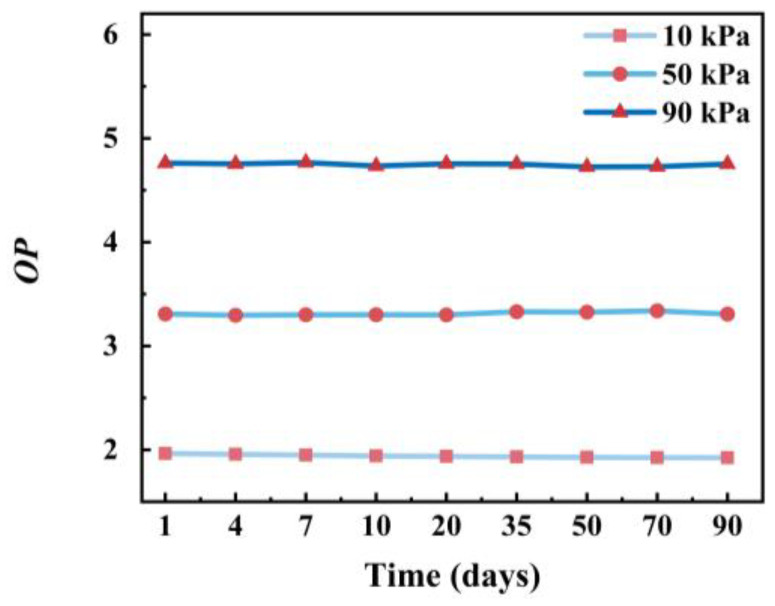
The *OP* values of the PtOEP/PDMS@Pine sensor measured at oxygen concentrations of 10 kPa, 50 kPa, and 90 kPa.

The oxygen measurement performance of the PtOEP/PDMS@Pine sensor was monitored over a 90-day period to assess its long-term stability. The *OP* values of the sensor at oxygen concentrations of 10 kPa, 50 kPa, and 90 kPa were monitored from day 1 to day 90. As illustrated in Figure 13, the *OP* values exhibited minimal variation under identical oxygen concentration conditions. These findings demonstrate that the sensor maintained stable oxygen detection performance over the 90-day evaluation period, confirming its capability for long-term application.

### 3.10. The Impact of Temperature on the Performance of the PtOEP/PDMS@Pine Sensor

**Figure 14:**
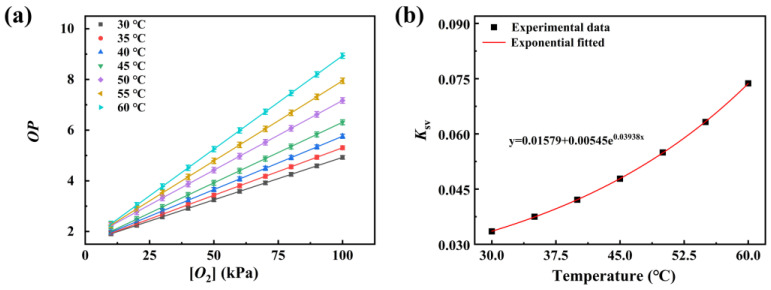
(**a**) The calibration curves of the PtOEP/PDMS@Pine sensor measured at different temperature in a range of 30–60 °C; (**b**) relationship between *K*sv values and temperature.

Temperature variations significantly affect the oxygen-sensing performance of optical oxygen sensors. In this study, we systematically investigated the mechanisms by which temperature influences sensor performance, as well as its specific effects. A thermal stage capable of simultaneous temperature control and ventilation was employed to investigate the influence of temperature on sensor performance. The relationship between the *OP* value of the sensor and the oxygen concentration at various temperatures was measured, as illustrated in Figure 14a. The results indicate that, at each temperature, the *OP* value exhibits a linear increase with rising oxygen concentration. By linear fitting the experimental data, the *K*sv values at variable temperatures were obtained, as presented in Figure 14b. This demonstrates that *K*sv increases with temperature elevation. This phenomenon can be attributed to the enhanced diffusion coefficient of oxygen molecules at higher temperatures, which increases the likelihood of interaction between indicator molecules and oxygen molecules, thereby enhancing *K*sv values. Furthermore, by applying an exponential fit to the *K*sv values obtained at different temperatures, the quantitative relationship between *K*sv and temperature (*T*) was established, as described in the following equation.

(6) $$K_{\mathrm{sv}}=0.016+0.005e^{0.0394T}$$

**Figure 15:**
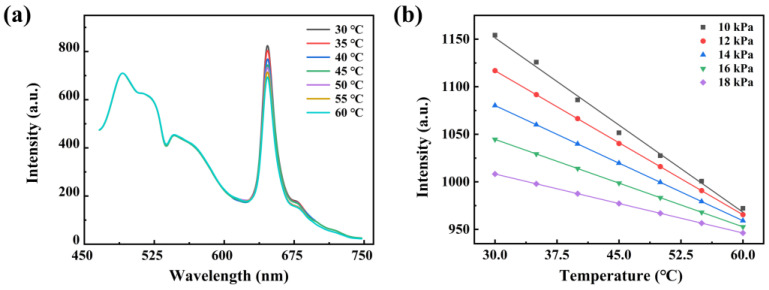
(**a**) The photoluminescence spectra of the PtOEP/PDMS@Pine sensor measured at different temperatures in the range of 30–60 °C; (**b**) the relationship between phosphorescence and temperature varies under different oxygen concentrations.

Simultaneously, the photoluminescence spectra of the sensor at different temperatures were also measured to investigate the influence of temperature on the luminescence characteristics of the sensor, as presented in Figure 15. As shown in the figure, the luminescence within the 450–600 nm range remains invariant with temperature changes, whereas the luminescence within the 600–700 nm range exhibits a decrease as the temperature increases. These findings suggest that the luminescence of the wood substrate remains stable across the selected temperature range, while the luminescence of PtOEP shows a significant reduction with increasing temperature. This implies that the reference signal *A*_1_ in the sensor is independent of temperature variations in a range of 30–60 °C. In contrast, the oxygen-sensitive signal *A*_2_ depends not only on oxygen concentration but also on temperature. By incorporating the relationship between *K*sv and temperature, the calibration curve of the original sensor was adjusted as below. According to this equation, the corresponding oxygen concentration can be precisely determined by measuring the *OP* value at a specified temperature.

(7) $$\frac{OP(t,T,O_{2})}{1.02-0.02e^{-2t}}=1.64+(0.016+0.005e^{0.0394T})[O_{2}]$$

### 3.11. The Impact of Humidity on the Performance of the PtOEP/PDMS@Pine Sensor

**Figure 16:**
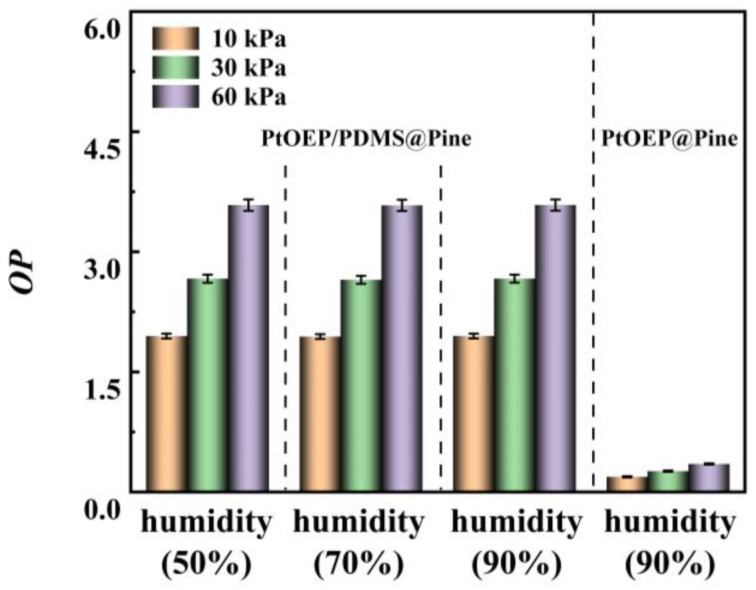
The *OP* values of the PtOEP/PDMS@Pine sensor at oxygen concentrations of 10 kPa, 30 kPa, and 60 kPa under different gas humidities.

In this study, the effect of humidity on sensor performance was further investigated. The *OP* values of the sensor at oxygen concentrations of 10 kPa, 30 kPa, and 60 kPa under humidity levels of 50%, 70%, and 90% were measured. Additionally, the *OP* values of the PtOEP@Pine sensor at an ambient humidity of 90% at the same oxygen concentrations was also measured for comparison. The experimental results are presented in Figure 16. From the figure, it can be observed that under identical oxygen concentration conditions, the *OP* values of the sensor remain stable across different humidity levels. This suggests that humidity does not affect the performance of the sensor. In contrast, at 90% humidity, the *OP* values of the PtOEP@Pine sensor decrease markedly under varying oxygen concentrations. This is because pine wood exhibits a strong tendency to absorb moisture, which reduces interactions between PtOEP molecules and oxygen molecules. The PtOEP/PDMS@Pine sensor, owing to its PDMS coating, mitigates the moisture absorption properties of pine wood, ensuring that the sensor’s performance remains unaffected by gas humidity and enabling its application in complex environments, including high-humidity conditions.

## 4. Conclusions

In this study, pine wood was employed for the first time as the supporting matrix for an oxygen sensor. Through a simple procedure, we successfully developed a PtOEP/PDMS@Pine optical oxygen sensor, which features a low cost and strong anti-interference capability. The sensor preserves the inherent porous structure of the wood, facilitating efficient oxygen transport. PtOEP and PDMS can fully infiltrate the entire 1 mm thickness of the sensor. Additionally, the sensor exhibits stable hydrophobicity, maintaining a water contact angle greater than 116° during continuous testing for 60 s, effectively addressing the issue of pine wood’s susceptibility to moisture absorption. The intrinsic luminescence of pine wood was utilized as the reference signal, while the luminescence of the oxygen-sensitive molecule PtOEP was used as the indication signal. By defining the ratio of the reference signal to the indication signal as the optical parameter (*OP*), it was found that *OP* exhibited a strong linear relationship with the oxygen concentration ([*O*_2_]) in the range of 10–100 kPa. The calibration curve was obtained (*OP* = 1.64 + 0.03 [*O*_2_]) under the optimal PtOEP/toluene concentration of 1 mM. Based on this linear relationship, the sensor enables precise measurement of the oxygen concentration within a range of 10–100 kPa. The experimental results demonstrated that both the reference signal (*A*_1_) and the indication signal (*A*_2_) increased with increasing laser power density, while the *OP* values remained stable. Moreover, under the same laser power density, multiple measurement results revealed that the fluctuation of the *OP* (~0.3%) value was significantly lower than that of *A*_1_ (~1%) and *A*_2_ (~2%), confirming the sensor’s excellent anti-interference performance.

Under alternating conditions of pure nitrogen and pure oxygen, the sensor displayed excellent recovery performance, with a rapid response to changes in oxygen concentration; the response and recovery times were determined to be 1.4 s and 1.7 s, respectively. The sensor demonstrates stable measurement performance over a 90-day long-term assessment period. Moreover, the sensing performance remains unaffected by humidity. The results of the light stability test demonstrate that both the reference signal and the oxygen indicator signal exhibit a decaying trend as the light exposure time increases, with decay rates of 0.01%/h and 0.2%/h, respectively. Furthermore, the impact of temperature on sensor performance has been comprehensively analyzed. By compensating for the calibration curve drift induced by these two factors, accurate measurement of the oxygen concentration in complex environments has been successfully realized.

## Data Availability

The data presented in this study are available on request from the corresponding author.

## Abbreviations

The following abbreviations are used in this manuscript:

PtOEP	Platinum(II) octaethylporphyrin
PDMS	Polydimethylsiloxane
*OP*	Optical parameter
[*O_2_*]	Oxygen concentration
*OS*	Optical signal
*T*	Temperature
